# Quantitative evaluation of vimentin expression in tumour stroma of colorectal cancer

**DOI:** 10.1038/sj.bjc.6603651

**Published:** 2007-02-27

**Authors:** C Y Ngan, H Yamamoto, I Seshimo, T Tsujino, M Man-i, J-I Ikeda, K Konishi, I Takemasa, M Ikeda, M Sekimoto, N Matsuura, M Monden

**Affiliations:** 1 Department of Surgery, Gastroenterological Surgery, Graduate School of Medicine, Osaka University, Osaka 565-0871, Japan; 2 Department of Pathology, Graduate School of Medicine, Osaka University, Osaka 565-0871, Japan; 3 Department of Pathology, School of Allied Health Science, Faculty of Medicine, Osaka University, Osaka 565-0871, Japan

**Keywords:** colorectal cancer, vimentin, prognosis

## Abstract

Recent studies have identified vimentin, a type III intermediate filament, among genes differentially expressed in tumours with more invasive features, suggesting an association between vimentin and tumour progression. The aim of this study, was to investigate whether vimentin expression in colon cancer tissue is of clinical relevance. We performed immunostaining in 142 colorectal cancer (CRC) samples and quantified the amount of vimentin expression using computer-assisted image analysis. Vimentin expression in the tumour stroma of CRC was associated with shorter survival. Overall survival in the high vimentin expression group was 71.2% compared with 90.4% in the low-expression group (*P*=0.002), whereas disease-free survival for the high-expression group was 62.7% compared with 86.7% for the low-expression group (*P*=0.001). Furthermore, the prognostic power of vimentin for disease recurrence was maintained in both stage II and III CRC. Multivariate analysis suggested that vimentin was a better prognostic indicator for disease recurrence (risk ratio=3.5) than the widely used lymph node status (risk ratio=2.2). Vimentin expression in the tumour stroma may reflect a higher malignant potential of the tumour and may be a useful predictive marker for disease recurrence in CRC patients.

Intermediate filament proteins form a dynamic cytoskeletal network in the cell to protect them against mechanical stress and to maintain cellular integrity (Clarke and Allan, 2002; Helfand *et al*, 2004). Vimentin is a type III intermediate filament characteristically found in cells of mesenchymal origin, that is fibroblast, chondrocytes, macrophages, and endothelial cells (Osborn, 1983). Vimentin was postulated to act as a scaffolding protein to stabilise connective tissues and cells, or in signal transduction (Herrmann *et al*, 2003; Eriksson *et al*, 2004). It was also reported to be involved in wound healing (Eckes *et al*, 2000) and lipid metabolism (Schweitzer and Evans, 1998).

Recently, an association between vimentin and tumour development, progression, and chemosensitivity was suggested by various gene profiling studies (Zajchowski *et al*, 2001; Mellick *et al*, 2002; Penuelas *et al*, 2005). Vimentin was selectively overexpressed in highly aggressive breast cancer cells (Zajchowski *et al*, 2001). It was also included, along with eight other genes, in a test that could sufficiently distinguish between invasive and noninvasive breast cancer (Nagaraja *et al*, 2006). Expression studies also indicated that vimentin overexpression in prostate cancer correlates with a more malignant phenotype (Lang *et al*, 2002; Singh *et al*, 2003). Furthermore, proteomic analysis identified the vimentin gene as one differentially expressed in colorectal cancer compared with the surrounding normal tissue (Alfonso *et al*, 2005). In regard to chemosensitivity, vimentin expression was higher in colon carcinoma cell clones resistant to doxorubicin, although vimentin alone did not confer resistance (Conforti *et al*, 1995). Similar findings were reported for a multidrug resistant breast cancer subline (Bichat *et al*, 1997).

In this study, we evaluated the significance of vimentin expression in colorectal cancer (CRC). Although colon cancer cells did not express vimentin, we found that stromal vimentin expression was quite abundant. Tumour stromal reaction is known to be dynamic. Difficulties in quantifying changes in the tumour stroma have limited the possibility of its utilisation in clinical settings. Here, we attempted semiquantification using computer-assisted imaging, and further analysed the correlation to clinicopathological factors and patients' survival.

## MATERIALS AND METHODS

### Patients

Vimentin expression was examined in a total of 142 CRC tissues of intermediate stages, that is, stage II (*n*=78) and stage III (*n*=63), based on the UICC TNM classification. The mean age of the patients was 62.5±9.5 years, with 62 women and 80 men. The tumours ranged in size from 0.8±12.0 cm (mean 5.1±1.8 cm), and were resected from either the colon (*n*=80) or rectum (*n*=62). The mean follow-up period was 66.1±29.4 months (range, 0.72–150.0 months). The overall 5-year survival rate was 82.4% and the 5-year disease-free survival rate was 76.8%. After surgery, stage III patients received 5-FU-based chemotherapy. Patients with stage II CRC had no chemotherapy unless the patient requested it. The study protocol was approved by the Human Ethics Review Committee of the Graduate School of Medicine, Osaka University.

### Histology

Tissue sections (4 *μ*m thick) were prepared from formalin-fixed paraffin-embedded blocks. Sections were stained with hematoxylin and eosin (H&E) solution, and reviewed by two pathologists from the Department of Pathology, Osaka University. The histological staging of tumours was as follows: well-differentiated adenocarcinoma (*n*=72), moderately differentiated adenocarcinoma (*n*=59), poorly differentiated adenocarcinoma (*n*=5), mucinous carcinoma (*n*=5), and signet ring cell carcinoma (*n*=1). The extent of stromal reaction was evaluated as extensive, moderate, and slight according to the criteria reported (Jass *et al*, 1986; Harrison *et al*, 1994). Tumour budding was evaluated based on the definition reported previously, and classified as high-grade budding and low-grade budding accordingly (Ueno *et al*, 2002). Characteristics of the tumour at the invasive margin were evaluated based on the diffuse infiltrative features of the tumour (Jass *et al*, 1987).

### Immunohistochemistry

Expression of vimentin was studied by immunohistochemistry. Immunostaining was performed using the Vectastain ABC peroxidase kit (Vector Labs, Burlingame, CA, USA), as described previously by our laboratories (Noura *et al*, 2002; Yamamoto *et al*, 2003). Tissues were sliced into 4 *μ*m sections, dewaxed in xylene, and rehydrated in decreasing concentrations of ethanol. Sections were subjected to endogenous peroxidase blocking in 1% H_2_O_2_ solution in methanol for 20 min and then to antigen retrieval treatment in 10 mM citrate buffer, pH 6.0, for 40 min in a water bath preheated to 95°C. Serum blocking was performed using 10% normal rabbit serum for 30 min at room temperature. This was followed by incubation with primary antibodies at 4°C overnight. Both the anti-vimentin (Novocastra, Newcastle, UK) and anti-proliferating cell nuclear antigen (PCNA) (PC10, Novocastra) monoclonal antibodies were diluted 1 : 50. Anti-CD34 (Novocastra) was used at 1 : 500 dilution. PCNA labelling was included as a quality control of tissue blocks. Secondary biotinylated anti-mouse antibody (BA2000, Vector Laboratories, Burlingame, CA, USA) was used at a dilution of 1 : 100 for 30 min at room temperature. Washing was performed using phosphate-buffered saline (PBS). Reaction product was visualised using 3,3′-diaminobenzidine (Wako Pure Chemical Industries, Osaka, Japan) as a chromogenic substrate. Sections were counterstained with hematoxylin, dehydrated, and mounted. For the negative control, nonimmunised mouse IgG (Vector Labs) was used in place of the primary antibodies.

### Computer-assisted imaging

**Figure 1:**
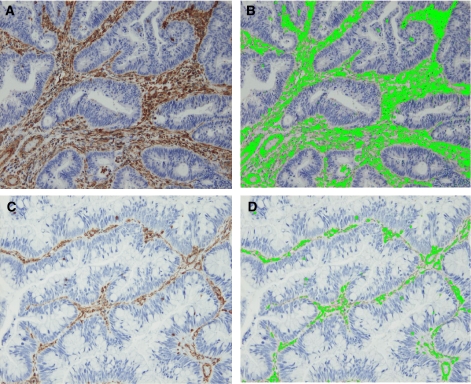
Representative sections of high vimentin expression (**A**) and low vimentin expression (**C**) in tumour stroma. Images were analysed based on the colour selection. Image analysis figures are shown (**B** and **D**). Area labelled (in fluorescent green) was calculated accordingly. Surface area was calculated as 22.0 and 3.3%.

The stained sections were viewed under a microscope equipped with a charge-coupled device (CCD) colour camera (Olympus Corp., Tokyo, Japan). We selected 10 fields in the ‘hot spots’ of positivity in each specimen at high power magnification. Photos were analysed using imaging processor Mac SCOPE software (Mitani Corp., Fukui, Japan), where the staining area (Figure 1) was calculated accordingly. Briefly, the investigator selected the brown-stained areas representative of vimentin expression on the photo. Vimentin expression in the colonic mucosal lymphocytes was used as internal positive control. The computer would then automatically detect the area with the same configuration on the photo and convert the data to a percentage of the total area in each field.

### Statistical analysis

Mean values were compared using the Student's *t*-test. Associations between discrete variables were assessed using the *χ*^2^ test. The Kaplan–Meier method was used to estimate tumour recurrence or death from CRC, and the log-rank test was used to examine statistical significance. A Cox proportional hazards model was used to assess the risk ratio under simultaneous contributions from several covariates. All statistical analyses were performed using the StatView J-5.0 programme (Abacus Concepts Inc., Berkeley, CA, USA). All data were expressed as the mean±s.d.. *P*-values of less than 0.05 were accepted as statistically significant.

## RESULTS

### Expression of vimentin in CRC tissue

Vimentin expression was detected in the tumour stroma region (Figure 1). Staining was intense and homogenous. No expression was observed in the normal colonic epithelial cells or tumour cells. Using computer-assisted image analysis, vimentin scores were determined (Figure 1). When 10 fields were analysed, vimentin expression varied widely, ranging from 1.7 to 24.1% (mean 8.8±4.3%). The quality of all tissue blocks was confirmed by intense staining for PCNA in the proliferative zone of normal colonic epithelium and germinal centres of lymphoid follicles.

### Vimentin expression and clinicopathological characteristics

Using the mean value of vimentin expression, 8.8%, as a cutoff point, the sample population was divided into high-expression (*n*=59) and low-expression groups (*n*=83). When vimentin expression was compared with the clinicopathological parameters listed in Table 1, an association was found between vimentin expression and age. Other factors were not associated with vimentin expression.

### Vimentin expression and histological characteristics at the tumour–stroma interface

Changes in the stroma may have an active role in the acquisition of invasive phenotype of tumour cells. We tested whether vimentin expression is associated with these histological characteristics at the tumour–stroma interface.

Tumours displaying diffuse infiltration pattern at the invasive front are deemed to be more aggressive. In this series, the 52.8% of the tumours exhibited this feature whereas the remaining 47.2% of the tumours lacked this feature. No association between this feature and vimentin expression was found.

Tumour budding was another histological change noted at the invasive front. This histological dedifferentiation phenomenon was observed in immature stroma, possibly promoting tumour progression (Ueno *et al*, 2004). Of the 142 CRC cases, only 27 (19.0%) showed high-grade tumour budding and the remaining 115 (81.0%) showed low-grade tumour budding. No significant association was found between tumour budding and vimentin expression.

We also compared vimentin expression with the extent of stromal response. In this series of CRCs, 24 tumours (16.9%) showed extensive stromal response, 95 (66.9%) showed moderate response, and 23 (16.2%) showed a slight response. No association was noted between this response and vimentin expression.

### Survival analysis

**Figure 2:**
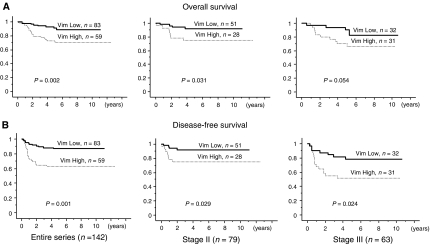
Survival curves were plotted using the Kaplan–Meier method for high vimentin (Vim High) expression and low (Vim Low) expression groups. (**A**) Overall survival. (**B**) Disease-free survival. Both end points were further analysed according to tumour staging (stages II and III).

For the overall and disease-free survival analysis, high vimentin expression was significantly associated with a shorter survival. For overall survival (Figure 2A), 5-year survival for the high-expression group was 71.2% compared with 90.4% for the low-expression group (*P*=0.002). Similarly, as shown in Figure 2B, the 5-year disease-free survival rate was 62.7% for the high-expression group compared with 86.7% in the low-expression group (*P*=0.001). When we further analysed the survival data for stage II (without lymph node metastasis) and stage III (with lymph node metastasis) CRC, we found that irrespective of the lymph node status, vimentin expression was significantly associated with a higher disease recurrence rate (Figure 2B). For stage II tumours, the disease-free survival rate for the high vimentin expression group was 71.4% compared with 92.3% in the low-expression group (*P*=0.029). Meanwhile, for stage III tumours, the disease-free survival for the high-expression group was 45.2% compared with 79.4% in the low-expression group (*P*=0.024). However, for overall survival (Figure 2A), an association was found only in stage II (75.0 *vs* 92.2%, *P*=0.031), but not in stage III (67.7 *vs* 87.5%, *P*=0.054). Considering that disease recurrence may provide a better understanding of clinical prognosis, further analyses were performed based on disease recurrence rather than overall survival (Andre *et al*, 2004).

Univariate survival analyses for other clinicopathological parameters and a few histological characteristics at tumour–stroma interface are summarised in Table 2. Of all parameters, lymph node metastasis status was of prognostic value, as expected. No other parameters showed significant prognostic value. Multivariate analysis of vimentin expression and other histopathological factors (Table 3) revealed that vimentin was an independent prognostic factor for CRC disease recurrence, with the high-expression group having a 3.5-fold greater risk of recurrence compared with the low-expression group. The risk ratio was also higher compared with lymph node status (relative risk of 2.2-fold). In addition, the diffuse infiltration characteristic at the invasive front was also shown to be an independent prognostic factor with a relative risk of 2.3-fold.

### Vimentin expression and microvascular density

Endothelial cells also display reactivity to anti-vimentin antibody. Therefore, we also evaluated endothelial cells using antibody against CD34. The total area stained for CD34 ranged from 0.09 to 2.42%, with a mean of 0.82%. CD34 staining accounted for less than 10% of the area staining for vimentin. We re-examined the prognostic value of vimentin expression after deducting the total area staining for CD34 to test whether microvascular density contributed to the prognostic significance of vimentin. Using the average mean value (7.96%) of vimentin after this adjustment as a cutoff point, a statistically significant difference (*P*=0.008) was still observed between the high-and low-expression groups.

## DISCUSSION

Tissue stroma consists of a variety of matrix substances such as interstitial collagen, fibronectin, elastin, and glycoaminoglycans and a variety of cell types including inflammatory cells, immune cells, fibroblasts, muscle, and vascular cells (Dvorak, 1986). Stromal microenvironment in tumour has a crucial role in tumour progression. It provides an interface between malignant cells and host tissues (Bissell and Radisky, 2001). Cumulative evidence suggests that the balance of host–tumour interdependency could modulate the phenotype of a tumour, and thus influence the outcome of the disease. However, appropriate markers to quantify the stromal reaction have yet to be determined.

Vimentin is ubiquitously expressed by cells of mesenchymal origin including fibroblasts, endothelial cells, smooth muscle cells, leucocytes, and some other cells (Dulbecco *et al*, 1983; Mor-Vaknin *et al*, 2003). In certain carcinomas such as breast cancer or melanoma, vimentin was upregulated in aggressive phenotypes in a phenomenon known as epithelial–mesenchymal transition (Brabletz *et al*, 2005). However, this phenomenon was not observed in CRC. In fact, in CRC, vimentin was specifically expressed in the stroma, but not in the tumour cells (Altmannsberger *et al*, 1982; von Bassewitz *et al*, 1982; Sordat *et al*, 2000). Thus, in this study we attempted to quantitate the expression of vimentin to verify the clinical value of the stromal response in CRC.

We found that vimentin expression in the tumour stroma was useful in identifying CRC patients with a poor prognosis. Increased stromal vimentin expression indicated a dynamic change in the tumour stroma during tumour progression. Previous attempts to evaluate the stromal response were based mostly on histological changes of the fibrous tissue in the stroma, including an evaluation of the relative amount of fibrous tissue or the pattern of stroma (Jass *et al*, 1986; Ueno *et al*, 2004). Results have been controversial with regard to prognosis. Some suggested a positive correlation, but others have suggested otherwise (Jass *et al*, 1986; Halvorsen and Seim, 1989; Harrison *et al*, 1994; Ueno *et al*, 2004). Nevertheless, these studies indicated that significant histological changes could be observed in the tumour stroma.

A significant part of the change could be attributed to fibroblastic changes, as suggested by other studies, as fibroblasts are the main cell population in tissue stroma. Our data suggest the possibility that stromal fibroblasts may indeed facilitate tumour progression, possibly invasion, and metastasis, leading to a higher rate of disease recurrence. Fibroblasts, being both activated by cytokines and at the same time producing cytokines or other soluble factors, were reported to modulate various aspects of tumour progression including proliferation or invasion (Vogetseder *et al*, 1989; Nakamura *et al*, 1997), angiogenesis (Orimo *et al*, 2001), or inhibition of cell death (Olumi *et al*, 1998). Vimentin expression is reportedly universally found in all types of fibroblasts (Skalli *et al*, 1989; Sappino *et al*, 1990). In comparing the extent of stromal reaction and vimentin expression in this study, however, our data suggest that fibrous tissue itself may not be sufficient to promote tumour progression. Collaboration with other factors in the stroma, including the cellular compartment consisting of lymphocytes and endothelial cells may be necessary to create a microenvironment favourable for tumour progression.

Other histological changes often observed in the tumour stroma are the appearance of lymphocytes. These infiltrating lymphocytes are known as tumour-infiltrating lymphocytes (TIL). Vimentin expression is also found in this group of cells. Thus, increased vimentin expression could also indicate increased numbers of TIL. Whether this group of cells protects the host against the tumour cells, or prevents a tumour-specific immune response, remains a controversial topic. A gradual increase in TIL was observed during melanoma tumorigenesis (Hussein *et al*, 2006). However, increased immune cells in CRC are reportedly associated with better survival (Pages *et al*, 2005). If indeed the increased vimentin is caused by this group of cells, our results would advocate that TIL suppress the immune response against the tumour. However, we note that recent studies have indicated that different subsets of TIL might have distinct roles in the tumour microenvironment (Yu and Fu, 2006).

As vimentin also stains endothelial cells, increased microvessels in these regions also caused an increase in overall vimentin expression in the stroma. As tumours grow, development of blood vessels becomes necessary to provide needed oxygen and nutrients. In CRC, the correlation between microvessel density and prognosis has been variable, with studies indicating both positive and negative correlations (Neal *et al*, 2006). In this series, we showed that although the single factor change of microvessel density did not provide any prognostic significance, the overall evaluation with vimentin had useful prognostic value to differentiate between high-risk and low-risk groups.

Furthermore, we found that the prognostic power of vimentin expression was better than that of lymph node metastasis. These data support the notion of vimentin as a novel tumour stromal prognostic marker in CRC. We also found that the prognostic power of vimentin was independent of lymph node status, as well as the stage of histological differentiation. These results support the proposal that stromal therapy may be a viable approach to CRC. Stromal therapy was proposed to be more flexible and applicable to a wider range of disease stages, as its target is dynamic (Liotta and Kohn, 2001). In hepatocarcinoma, chemotherapy was demonstrated to be more effective, if therapies against the underlying fibrosis were also employed (Friedman *et al*, 2000; Bilimora *et al*, 2001). It is also of interest that stromal markers, such as vimentin in this study, may be useful in monitoring stromal therapies. Targeting the tumour as an organ would be more effective than targeting the tumour alone.

Here, we provide clinical evidence of stromal response, as evaluated by vimentin expression, as a prognostic indicator for poor prognosis in CRC patients irrespective of lymph node status. Vimentin staining allows an evaluation of overall stromal changes that include fibroblastic changes, microvessel density, infiltrating lymphocytes, and possibly other stromal changes yet to be identified. We note, however, that although the results presented here may be useful as a biological marker, they might not specifically reflect the biological nature of cancer (Nishio *et al*, 2001). Further assessment of other stromal reaction markers should allow a better understanding of more specific interactions between tumour cells and the microenvironment. A larger scale prospective study will be necessary to verify the prognostic significance of this stromal marker.

## Figures and Tables

**Table 1:** Vimentin expression and patient characteristics

	**Vimentin expression**		
**Clinicopathological characteristic**	**High**	**Low**	***P*** **-value**
Age	64.4±8.6	61.1±9.9	**0.037**
Tumour size	0.3±2.0	5.0±1.7	0.348
			
*Gender*			
Male	32	48	0.670
Female	27	35	
			
*Tumour site*			
Colon	36	44	0.343
Rectum	23	39	
			
*Degree of differentiation*			
Well	27	45	0.321
Mod./poor	32	38	
			
*Depth of invasion*			
mp	5	8	0.812
ss	54	75	
			
*Lymph node metastasis*			
Absent	28	51	0.098
Present	31	32	
			
*No of lymph nodes involved*			
0	28	51	0.167
1–3	20	24	
⩾4	11	8	
			
*Lymphovascular invasion* a			
Absent	24	32	0.799
Present	35	51	

Well=well-differentiated adenocarcinoma, Mod.=moderately differentiated adenocarcinoma, Poor=poorly differentiated carcinoma (this category included five cases of poorly differentiated adenocarcinoma, five cases of mucinous carcinoma and one case of signet ring cell carcinoma), mp=muscularis propria; ss=subserosa.

a Lymphatic invasion was determined by the presence of tumour cells in lymphatic ducts. Bold value is statistically significant (*P*<0.05).

**Table 2:** Univariate survival analysis (disease-free survival)

**Characteristics**	**Category**	***n***	**5-year survival**	***P*** **-value**
Vimentin	<8.8%	83	86.7	**0.001**
	⩾8.8%	59	62.7	
Age (years)	<62	64	76.7	0.986
	⩾62	78	76.9	
Tumour size (cm)	⩾5.1	71	76.1	0.881
	<5.1	71	77.5	
Gender	Male	80	78.8	0.631
	Female	62	74.2	
Tumour site	Colon	80	73.8	0.420
	Rectum	62	80.7	
Degree of differentiation	Well	72	80.6	0.292
	Mod./poor	70	72.9	
Depth of invasion	mp	13	69.2	0.376
	ss	129	77.5	
Lymph node metastasis	Absent	79	86.1	**0.004**
	Present	63	65.1	
Lymphovascular invasion^a^	Absent	56	80.4	0.291
	Present	86	74.4	
Diffuse infiltration	Absent	67	83.6	0.051
	Present	79	70.7	
Tumour budding	Low grade (<10)	120	75.0	0.238
	High grade (⩾10)	22	86.4	
Stromal reaction	Extensive	24	75.0	0.884
	Moderate/slight	118	77.1	

mp=muscularis propria; ss=subserosa.

a Lymphatic invasion was determined by the presence of tumour cells in lymphatic ducts. Bold values are statistically significant (*P*<0.05).

**Table 3:** Multivariate analysis (disease-free survival)

	***P*-value**	**Risk ratio**	**Confidence interval**
**Vimentin (high: low)**	**0.001**	**3.45**	**1.65**–**7.22**
Lymphovascular invasion (present: absent)	0.567	1.24	0.59–2.62
**Diffuse infiltration (present: absent)**	**0.047**	**2.29**	**1.01**–**5.18**
Tumour budding (high grade: low grade)	0.340	0.62	0.24–1.65
**Lymph node metastasis (present: absent)**	**0.043**	**2.20**	**1.02**–**4.72**
Depth of invasion (ss: mp)	0.445	0.64	0.20–2.02
Stromal reaction (extensive: moderate/slight)	0.875	1.08	0.42–2.78
